# Syndromic Surveillance in Public Health Emergencies: A Systematic Analysis of Cases Related to Exposure to 2023 Floodwaters in Romagna, Italy

**DOI:** 10.3390/healthcare12171760

**Published:** 2024-09-03

**Authors:** Marco Montalti, Marco Fabbri, Raffaella Angelini, Elizabeth Bakken, Michela Morri, Federica Tamarri, Chiara Reali, Giorgia Soldà, Giulia Silvestrini, Jacopo Lenzi

**Affiliations:** 1 Unit of Hygiene and Public Health Forlì-Cesena, Department of Public Health, Romagna Local Health Authority, 47522 Cesena, Italy; 2 Department of Medical and Surgical Sciences, University of Bologna, 40126 Bologna, Italy; 3 Unit of Hygiene and Public Health Ravenna, Department of Public Health, Romagna Local Health Authority, 48121 Ravenna, Italy; 4 Unit of Hygiene and Public Health Rimini, Department of Public Health, Romagna Local Health Authority, 47624 Rimini, Italy; 5 Unit of Environmental Epidemiology, Institute of Environmental Medicine, Karolinska Institutet, 171 77 Stockholm, Sweden; 6 Unit of Hygiene, Public Health, and Medical Statistics, Department of Biomedical and Neuromotor Sciences, University of Bologna, 40126 Bologna, Italy

**Keywords:** flood, syndromic surveillance, water contamination, public health preparedness and response, early warning system

## Abstract

Background: In May 2023, Romagna, Italy, faced a devastating flood resulting in 16 fatalities, forced displacement of 26,000 citizens, and significant economic losses. Due to potential water contamination, implementing public health strategies became imperative for the Local Health Authority to mitigate the health consequences, analyze the flood’s impact on the local population’s health, and detect early anomalies requiring timely public health interventions. Methods: Between June and July 2023, general practitioners who were part of the RespiVirNet surveillance network completed weekly structured forms. These forms collected data on individuals exposed or not to floodwaters and clinical syndromes. Rates per 1000 resident population aged > 14 were stratified by district, week of observation, and symptomatology. Missing data were addressed by imputation using second-order autoregressive modeling. Results: An incidence of 3.52 syndromes potentially related to flood water exposure per 1000 individuals (95% CI 2.82–4.35) was estimated. Ravenna, the city most affected by the flood, recorded the highest rate (6.05 per 1000, 95% CI 4.59–7.82). Incidence decreased in the weeks post-event. Anxiety, or trauma and stress symptoms, exhibited higher rates among the exposed, diminishing over weeks. The incidence for the non-exposed (12.76 per 1000, 95% CI 10.55–15.29) showed no significant territorial differences compared to the exposed ones. Conclusions: Syndromic surveillance provided timely information on the flood’s health impact, revealing a higher incidence of individual syndromes among the non-exposed. This study contributes to guiding the implementation of future public health preparedness and response strategies for populations facing similar natural disasters.

## 1. Introduction

In May 2023, a catastrophic flood affected the northern Italian region of Emilia-Romagna. On 2 May 2023, the civil protection authorities issued the first red alert for hydraulic risk [1]. Subsequently, the region experienced unprecedented rainfall, with precipitation levels exceeding 200 mm within 48 h in certain areas. The eastern part of the region, notably the Forlì province, bore the brunt of these heavy rainfalls, with precipitation surpassing 300 mm on 15–17 May [2]. By 17 May, a total of 23 rivers and streams had overflowed their banks, with numerous others nearing critical levels. Approximately 350 million m^3^ of water from rivers accumulated over an area of about 540 km^2^, covering entire cities and villages with water and mud for days [3]. Consequently, more than 26,000 individuals were displaced from densely populated zones, necessitating the urgent implementation of disaster-related public health measures [4]. In the province of Ravenna, 400 patients hospitalized in acute care and socio-sanitary facilities required evacuation and relocation [5].

Due to potential water contamination and environments conducive to pathogen growth, implementing public health strategies by the Local Health Authority became imperative to mitigate the health consequences associated with the flood [6]. In the days following the flood, Public Health Departments undertook several critical actions. These included identifying safe zones for waste disposal, managing livestock impacted by the floodwaters, and administering vaccinations for tetanus and Hepatitis A. The vaccination efforts were tailored to individuals based on their specific risk levels and types of exposure, particularly for those involved in home recovery and waste disposal activities or those exposed to standing water. These individuals included not only public service employees and volunteers but also numerous unpaid and often untrained individuals in environmental event management and restoration activities.

On 26 May 2023, the Public Health Department of Romagna’s Local Health Authority (LHA) disseminated a leaflet containing instructions for flooded residents and individuals involved in home recovery and waste disposal activities to prevent potential accidents, injuries, and infectious disease outbreaks and to promote vaccinations [7].

Several studies have investigated the relationship between health outcomes and floods, finding a positive correlation with heavy rainfall events, particularly during spring and summer [8,9]. Common health impacts of floods include gastrointestinal infections, skin ailments, and respiratory issues due to sewage system failures, compromised hygiene, overcrowding, and contaminated water sources [10,11].

Infectious disease risks following floods are not the most significant threats to the health and well-being of flood-affected communities. The literature also highlights several other health risks, including disruption of healthcare services (particularly for chronic care), environmental hazards (e.g., carbon monoxide poisoning, exposure to hazardous chemicals), and psychological stress, which may lead to long-term health effects and an increase in all-cause mortality [12]. Exposure to natural disasters such as floods can indeed have adverse effects on mental health, with reports of psychological distress among affected populations [13,14].

In a recent systematic review conducted in 2021 by Weilnhammer et al., which included studies from high-income European countries, it was found that although floods in the examined contexts might not be directly associated with mortality, they were linked to mental health morbidity. This included long-term sequelae such as depression, anxiety, and post-traumatic stress disorder [11]. However, with climate change significantly increasing the frequency of river flooding in both high-income and low- to middle-income countries in recent years [15], the flood event in Libya in September 2023 highlighted how the health impacts of floods can vary depending on healthcare preparedness and disaster management protocols [16]. Indeed, in this low-income country, which was already grappling with a complex set of challenges including military conflicts, the flood resulted in 4333 deaths. This underscored the urgent need for a re-evaluation of disaster management protocols, particularly for low-resource settings and conflict-afflicted zones, to ensure effective emergency response preparedness and management of long-term health implications [16,17].

Although extreme events provide valuable insights into adaptation strategies against river flooding, there is still limited evidence regarding the health effects of floods and the effectiveness of public health interventions [14,18].

In a July 2021 Rapid Risk Assessment, the European Center for Disease Prevention and Control (ECDC) recommended several public health interventions in response to extreme rainfall and catastrophic floods in western Europe. These included ensuring prompt access to clean water for affected communities, guaranteeing the availability of vaccinations, implementing water management plans to minimize the risk of Legionella growth, and monitoring and potentially treating flooded areas to prevent increases in mosquito populations. Effective risk communication with affected communities was also emphasized, recommending a structured approach to clearly deliver key messages and address the needs of the affected populations [19].

Finally, the ECDC advised affected countries and regions to consider implementing syndromic and event-based surveillance systems to rapidly detect and respond to potential outbreaks. Suspected cases of infectious diseases, including clusters of respiratory and gastrointestinal symptoms, rashes, etc., from a predefined list, should be reported immediately to local and national public health authorities to prompt a swift response [19].

The aim of this study was to document disease patterns and symptom clusters associated with flood water exposure in the Romagna region using a syndromic surveillance approach. We aimed to identify potential increases in incidence rates and variations across different areas within the region for infectious diseases and mental health outcomes.

## 2. Materials and Methods

Between 12 June and 9 July 2023, the Department of Public Health implemented a surveillance and early warning system by collecting weekly reports from family doctors over four consecutive weeks. The initiation of syndromic surveillance data collection was delayed relative to the flood event due to the simultaneous implementation of comprehensive public health measures aimed at mitigating its health impacts. Moreover, it was necessary to initiate this surveillance system de novo, starting with the creation of a weekly data collection form.

Consequently, a retrospective collection of aggregated data from the same family doctors was conducted concerning cases occurring among individuals exposed in the initial weeks following the flood and preceding the onset of syndromic surveillance. Data on the same syndromes targeted in the subsequent syndromic surveillance were retrospectively gathered for the month preceding flood surveillance initiation (16 May to 11 June 2023), facilitated through a specially designed online form completed retrospectively by family doctors.

Regarding weekly syndromic surveillance, family doctors compiled weekly reports using a surveillance form. These forms gathered week-by-week information on specific symptoms associated with exposure to floodwaters in the Romagna region.

### 2.1. Population

The Romagna LHA, covering an area of approximately 5000 square km^2^, serves a population of 1,124,896 residents as of 1 January 2024. To implement the surveillance, it was decided to involve RespiVirNet, a network of 24 sentinel family doctors coordinated by the Italian National Institute of Health for monitoring cases of influenza-like syndromes and respiratory viruses [20]. The operational protocol of the RespiVirNet network mandates the annual recruitment of a sufficient number of sentinel doctors to achieve a coverage of at least 4% of the population for each age group. However, due to the significant impact of the flood on certain specific and restricted areas of the Romagna LHA, it was decided to also involve the 10 family doctors operating in the areas most affected by the flood, integrating them into the existing RespiVirNet network. Consequently, the 34 physicians involved in the surveillance were representative of areas exposed to different consequences of the flood: areas where water rapidly drained, leaving mud behind; areas where water remained stagnant for an extended period; and areas with the flow of floodwaters.

### 2.2. Forms and Data Collection

The paper-based form used to collect weekly prospective data required each row to be filled out per patient, allowing family doctors to indicate the characteristics (age and gender) of the individuals visited, the exposure (contact with floodwaters, flooded house, house submerged for 3 days or more, involvement in cleaning and restoration activities) or non-exposure, and the syndromes by checking the respective boxes. Due to the absence of standardized forms at the Italian or European level, the form in question was derived and adapted from the “Natural Disaster Morbidity Surveillance Line List (Interim)” form issued by the Center for Disease Control [21]. The form was translated into Italian and adapted, retaining only the syndromes that potentially occur following a flood [22]: dermatological syndromes (primary skin infections and secondary infections of lesions), gastrointestinal syndromes (diarrhea, vomiting, abdominal cramps), respiratory syndromes (congestion, cough, suspected pneumonia), ophthalmological syndromes (conjunctivitis), otological syndromes (otitis), body temperature exceeding 37.5 °C for more than 48 h, and anxiety or trauma and stress-related symptoms (insomnia, restlessness, anxiety requiring medication). Additionally, a notes section was available for physicians to report relevant situations not covered by the predetermined entries on the form. The form used in this syndromic surveillance is available in the Supplementary File.

To collect retrospective data from 16 May to 11 June, a simplified version of the form was used to reduce the risk of recall bias. This version allowed family doctors to report only the number of syndromes among exposed versus non-exposed individuals, without stratification by week or patient demographics.

In the week before the syndromic surveillance started, an introductory online meeting was organized with all 34 of the family doctors participating in the study. During the call, a brief overview of the project organization and methodology was provided, and the weekly data collection forms were discussed and presented, along with a guide for completion. Procedures for retrospectively reporting aggregated data with the specially designed online form from previous weeks were also shared. Additionally, dedicated phone lines were made available for addressing any needs or concerns that the participating LHA physicians might have had while filling out the forms at the Public Health Department locations in the four districts of Romagna. Every Monday, for 4 weeks, a reminder email was sent to all participating family doctors involved in the syndromic surveillance project. Additionally, doctors who did not submit the forms within the agreed-upon timeframe were contacted by phone by Public Health physicians to ascertain their willingness to continue participating in the project. The forms for the 4 weeks were scanned and submitted via email by the family doctors every week, and the data were then manually entered into a computer system by personnel from the Public Health Department. Data management of the Local Health Authority was performed following the General Data Protection Regulation of the European Union.

### 2.3. Statistical Analysis

Individual cases of waterborne diseases per 1000 population reported between 12 June and 9 July 2023 were stratified by district (Ravenna, Forlì, Cesena, Rimini), week, and symptomatology. The denominators for these calculations consisted of the patient panel sizes of the family doctors who participated in the surveillance program. The 95% confidence intervals (CIs) of rates were calculated using exact Poisson limits (Poisson means).

Eighteen out of 34 family doctors with extensive missing values (<2 weekly reports between 12 June and 9 July) or no information about patient panel size were excluded from the analysis. If two or three weekly reports were available, the missing values were imputed for each physician with the predictions obtained from a second-order autoregressive Poisson regression model. The stability of the forecasts obtained by assuming a linear trend was confirmed in a sensitivity analysis after including restricted cubic spline terms in the model.

Aggregate data on waterborne diseases that occurred in the preceding month were also displayed for comparison. Furthermore, data pertaining to the same diseases among individuals not exposed to contaminated water between 12 June and 9 July 2023 were reported and analyzed. A limited number of family doctors, specifically 14 for aggregated data concerning the initial post-flood period, and 6 for data on non-exposed patients, provided this information. District-specific rates were displayed with the aid of choropleth maps by partitioning the range of values into intervals of equal width. All analyses were carried out with Stata 17 (StataCorp. 2021. Stata Statistical Software: Release 17. College Station, TX, USA: StataCorp LLC).

## 3. Results

The overall data generated by the 16 family doctors included in the final analysis, encompassing all LHA districts, indicate that 86 individuals exposed to contaminated water over the age of 14 out of a total population of 24,433 (3.52 per 1000) experienced symptoms during the study period, which spanned from 12 June to 9 July 2023. The weekly average of reported cases was 21.5 (Table 1).

Upon closer examination of district-specific data, variations became apparent. Ravenna exhibited the highest cumulative rate at 6.05 per 1000 population (95% CI 4.59–7.82), followed by Forlì at 2.50 per 1000 population (95% CI 1.45–4.00). Weekly estimates for individual districts displayed fluctuations, highlighting the temporal dynamics of the health impact.

Nevertheless, a common trend was observable across all LHA districts in the Romagna region, which consistently recorded higher case estimates during the initial weeks of the syndromic surveillance, followed by a gradual incidence decrease (Table 1).

**Figure 1:**
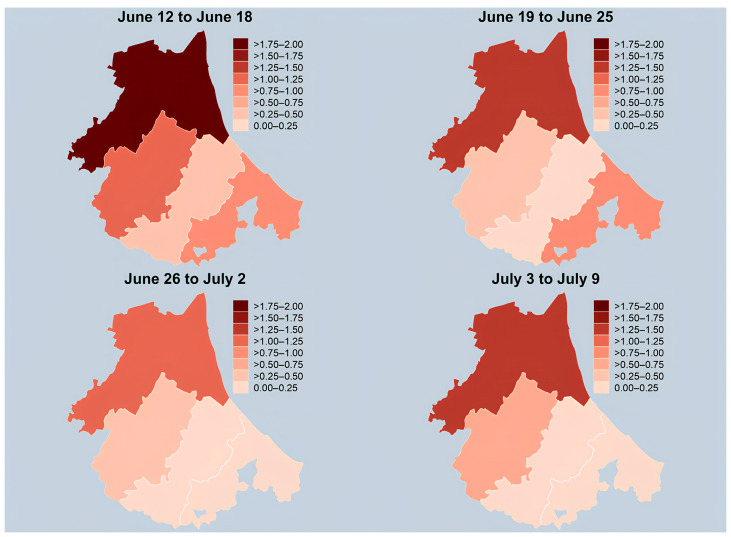
Symptomatic people aged >  14 years exposed to contaminated water (× 1000 population) during the 2023 Romagna floods, by district of residence (Ravenna, Forlì, Cesena, Rimini) and week (12 June to 9 July 2023). Notes: A total of 16 family doctors were included in the analysis (six in Ravenna, four in Forlì, three in Cesena and three in Rimini).

Figure 1 offers a comprehensive graphical overview of the health impact caused by the floodwaters, enabling a nuanced understanding of both the timing and geographical patterns of the reported cases.

Considering individuals not exposed to contaminated water, as reported by family doctors in the four districts during the same period, it was observed that 117 individuals over the age of 14, out of a total of 9170 (12.76 per 1000 population), experienced symptoms, with a weekly average of 29.3 cases (Table 2). Similarly, the district-specific breakdown revealed variations, with Rimini exhibiting the highest cumulative rate at 16.16 per 1000 population (95% CI 11.65–21.84). Weekly averages for individual districts fluctuated across different weeks, though without allowing for the identification of any specific trends in the estimates.

In comparing syndromic surveillance data gathered from 12 June to 9 July with retrospectively collected and aggregated data from the first month following the flood (16 May–11 June), a noticeable decrease in symptom rates among exposed individuals during the latter period compared to the former was apparent, with variations observed among districts (Table 3). Specifically, there was a decrease from 0.80 per 1000 population to 0.33 for dermatological infections, from 2.26 to 1.06 for gastrointestinal infections, from 0.85 to 0.20 for conjunctivitis, from 0.61 to 0.29 for otitis, from 1.23 to 0.49 for febrile symptoms, and from 1.70 to 0.16 for psychological issues. Only respiratory syndromes (chest congestion, cough, or suspected pneumonia) showed an increase between the two observation periods. Notably, Ravenna consistently reported higher estimates for all observed symptoms during both time periods.

Finally, upon analyzing the rates of individual symptoms among those who had not been exposed to floodwaters, it was evident that the estimates were indeed higher for almost all symptoms compared to those calculated for the exposed population (Table 4). However, the exposed population showed higher rates of psychological issues, such as insomnia, restlessness, and anxiety, compared to the population not exposed to floodwaters.

## 4. Discussion

This study explored the health impacts of exposure to contaminated water following a flood event in the Romagna region. Floods are among the most prevalent natural disasters globally, and their frequency is expected to increase due to climate change [23].

Analysis of syndromic surveillance data collected from general practitioners provided valuable insights into the prevalence and temporal patterns of various symptoms, facilitating the calculation of incidence rates for both exposed and unexposed individuals. Significantly, rates of dermatological, gastrointestinal, respiratory, conjunctivitis, otitis, and fever symptoms were higher among unexposed patients compared to those exposed to floodwaters. However, a notable exception was a higher incidence of psychological issues such as insomnia, restlessness, and anxiety among individuals exposed to floodwaters. This finding appears surprising given that the scientific literature suggests potential increases in the incidence rates of infectious diseases following floods [11]. Nonetheless, some arguments could elucidate this phenomenon. First, individuals exposed to floodwaters, whether through residence in flooded areas or participation in recovery efforts, may have had limited access to primary healthcare providers, potentially seeking medical attention solely for more severe symptoms. Second, it is conceivable that vulnerable individuals were protected during the pre-flood alert phase, thereby experiencing reduced exposure to floodwaters compared to the general population. These factors likely contributed to the observed lower rates of syndromes among the exposed.

Supporting the reliability of syndromic surveillance data collection, a decrease over the following weeks was observed exclusively among individuals exposed to floodwaters, with higher rates noted in the districts of Romagna most severely affected by the flood. Specifically, rates of dermatological, gastrointestinal, conjunctivitis, otitis, fever symptoms, and psychological problems declined among the exposed individuals. In contrast, the rates for unexposed individuals remained stable throughout this time. The declining trend among the exposed is not unexpected, given the transition from the emergency phase to recovery in subsequent weeks. This transition was characterized by reduced exposure intensity and increased availability of protective measures.

Ravenna, the area most affected by the flood, consistently reported higher symptom rates among exposed individuals. This regional disparity is due to the extensive coverage of the LHA, with different territories being impacted to varying degrees by the weather event. For instance, in Rimini, which was less severely affected, incidence rates were significantly lower compared to those in Ravenna. Furthermore, respiratory infections were the only persistent symptoms in Ravenna, likely attributed to prolonged flooding duration and weeks of recovery efforts, influencing these estimates. The association between respiratory and gastrointestinal symptoms with floods was also demonstrated in a 2019 study in the Netherlands, identifying post-flooding cleaning operations as a risk factor for acute respiratory infections [24].

Conversely, rates among unexposed LHA individuals not only remained constant over time but also exhibited uniformity across all territories, suggesting good reliability of the syndromic surveillance implemented.

A particularly significant finding was the above-mentioned higher rates of onset of insomnia, agitation, and anxiety requiring medication among both exposed and unexposed populations, especially in the first month post-flood. Mental health is profoundly affected by natural disasters such as floods, earthquakes, and hurricanes, with a higher incidence in low- and middle-income countries where resources to address these issues are limited [18]. During the winter of 2013/14, following severe flooding in England, a cross-sectional analysis of flood-affected individuals revealed a high prevalence of psychological morbidity among those directly flooded and those experiencing essential service disruptions, highlighting the significant and prolonged impacts of floods [25]. Another study in the UK previously measured increased psychological distress among adults affected by floods, with a fourfold risk for those with home damage [26]. Although few studies examine the impact of floods on children’s mental health, available research indicates significant behavioral changes, such as aggression and sleep and bladder problems [27,28,29].

The syndromic surveillance conducted in the aftermath of the flood did not identify any alerts. The identification of any disproportionately occurring syndromes would have been highly advantageous for bolstering public health interventions aimed at preventing and controlling infectious diseases during this critical period. Moreover, it would have necessitated a more thorough monitoring of diagnoses made in hospital settings and laboratory reports, which were already routinely checked through traditional surveillance systems.

Due to climate change, extreme rainfall events will occur more frequently, leading to more people being exposed to pluvial flood water in urban areas in countries like Italy [23,30]. A key attribute of this syndromic surveillance system is its flexibility and simplicity, enabling near real-time response to different public health incidents at national and local levels, and contributing to monitoring seasonal syndromes or those following natural disasters [31].

Despite its usefulness, syndromic surveillance conducted in Romagna faced several significant limitations that must be addressed. Firstly, due to the lack of prior planning and organization, as well as the fact that the personnel responsible for establishing surveillance were the same individuals involved in supporting the population and administering vaccinations, several weeks were needed to produce data sheets, engage family doctors, and activate a network of family doctors within the Public Health Department for data collection and analysis. This delay necessitated the retrospective collection of data for the first month following the flood, asking family doctors only to provide counts of waterborne diseases without detailed patient information, except for exposure to contaminated water. This approach was chosen to minimize the risk of recall bias and encourage participation in the survey, but it prevented us from calculating the rates of symptomatic individuals during this period. Nonetheless, the data collected appeared plausible and consistent with the results from the prospective survey. Additionally, there was limited adherence by family doctors to the syndromic surveillance, and their discontinuation of participation during the study posed significant challenges. However, the availability of the RespiVirNet network, despite its different objectives, served as a facilitating factor due to its personnel being accustomed to communicating and working together. Conducting syndromic surveillance requires additional effort and may be perceived as an added burden compared to routine or emergency activities. Although the population denominator (family doctors’ patients) was substantial, allowing for very narrow confidence intervals in syndrome incidence estimates, the case population was too small to stratify by age and gender.

Finally, and most importantly, the use of different numerators for exposed and unexposed individuals, while maintaining the same denominators, precluded definitive estimates of the flood’s effect size. Further refinement in planning, organization, and family doctors’ engagement is essential for more accurate and reliable syndromic surveillance in future studies.

## 5. Conclusions

In conclusion, this study revealed some unexpected results, such as the lower-than-anticipated incidence rates of most syndromes among exposed individuals and a notable increase in anxiety, trauma, and stress-related symptoms. The study underscored the advantages of the syndromic surveillance system, including its flexibility and simplicity, which allowed for near real-time monitoring and response. However, it also highlighted critical limitations in planning, data collection, and physician participation. Addressing these issues is essential for improving the accuracy and reliability of syndromic surveillance during future flood events, especially as climate change leads to more frequent and severe incidents. Implementing a well-established, ready-to-use syndromic surveillance system at the national or local level could help ease public concerns, detect early warning signs of health issues, and enhance response and preparedness efforts.

## Figures and Tables

**Table 1:** Symptomatic people aged >  14 years exposed to contaminated water during the 2023 Romagna floods, overall and by district of residence and week (12 June to 9 July 2023).

	Symptomatic	Rate × 1000 Population	
	Individuals	Estimate	95% CI
All (*n* = 24,433)			
Week 1 (12 June to 18 June)	30	1.23	0.83, 1.75
Week 2 (19 June to 25 June)	21	0.86	0.53, 1.31
Week 3 (26 June to 2 July)	16	0.65	0.37, 1.06
Week 4 (3 July to 9 July)	19	0.78	0.47, 1.21
Weeks 1–4 (12 June to 9 July)	86	3.52	2.82, 4.35
Weekly avg. (12 June to 9 July)	21.5	0.88	0.70, 1.09
Ravenna (*n* = 9585)			
Week 1 (12 June to 18 June)	18	1.88	1.11, 2.97
Week 2 (19 June to 25 June)	14	1.46	0.80, 2.45
Week 3 (26 June to 2 July)	12	1.25	0.65, 2.19
Week 4 (3 July to 9 July)	14	1.46	0.80, 2.45
Weeks 1–4 (12 June to 9 July)	58	6.05	4.59, 7.82
Weekly avg. (12 June to 9 July)	14.5	1.51	1.15, 1.96
Forlì (*n* = 6809)			
Week 1 (12 June to 18 June)	7	1.03	0.41, 2.12
Week 2 (19 June to 25 June)	3	0.44	0.09, 1.29
Week 3 (26 June to 2 July)	3	0.44	0.09, 1.29
Week 4 (3 July to 9 July)	4	0.59	0.16, 1.50
Weeks 1–4 (12 June to 9 July)	17	2.50	1.45, 4.00
Weekly avg. (12 June to 9 July)	4.3	0.62	0.36, 1.00
Cesena (*n* = 4171)			
Week 1 (12 June to 18 June)	2	0.48	0.06, 1.73
Week 2 (19 June to 25 June)	1	0.24	0.01, 1.34
Week 3 (26 June to 2 July)	1	0.24	0.01, 1.34
Week 4 (3 July to 9 July)	1	0.24	0.01, 1.34
Weeks 1–4 (12 June to 9 July)	5	1.20	0.39, 2.80
Weekly avg. (12 June to 9 July)	1.3	0.30	0.10, 0.70
Rimini (*n* = 3868)			
Week 1 (12 June to 18 June)	3	0.78	0.16, 2.27
Week 2 (19 June to 25 June)	3	0.78	0.16, 2.27
Week 3 (26 June to 2 July)	0	0.00	0.00, 0.95
Week 4 (3 July to 9 July)	0	0.00	0.00, 0.95
Weeks 1–4 (12 June to 9 July)	6	1.55	0.57, 3.38
Weekly avg. (12 June to 9 July)	1.5	0.39	0.14, 0.84

Notes: A total of 16 family doctors were included in the analysis (six in Ravenna, four in Forlì, three in Cesena and three in Rimini). Abbreviations: CI, confidence interval.

**Table 2:** Symptomatic people aged >  14 years not exposed to contaminated water during the 2023 Romagna floods, overall and by district of residence and week (12 June to 9 July 2023).

	Symptomatic	Rate × 1000 Population	
	Individuals	Estimate	95% CI
All (*n* = 9170)			
Week 1 (12 June to 18 June)	30	3.27	2.21, 4.67
Week 2 (19 June to 25 June)	27	2.94	1.94, 4.28
Week 3 (26 June to 2 July)	35	3.82	2.66, 5.31
Week 4 (3 July to 9 July)	25	2.73	1.76, 4.02
Weeks 1–4 (12 June to 9 July)	117	12.76	10.55, 15.29
Weekly avg. (12 June to 9 July)	29.3	3.19	2.64, 3.82
Ravenna (*n* = 3209)			
Week 1 (12 June to 18 June)	6	1.87	0.69, 4.07
Week 2 (19 June to 25 June)	5	1.56	0.51, 3.64
Week 3 (26 June to 2 July)	15	4.67	2.62, 7.71
Week 4 (3 July to 9 July)	10	3.12	1.49, 5.73
Weeks 1–4 (12 June to 9 July)	36	11.22	7.86, 15.53
Weekly avg. (12 June to 9 July)	9.0	2.80	1.96, 3.88
Forlì (*n* = 3362)			
Week 1 (12 June to 18 June)	14	4.16	2.28, 6.99
Week 2 (19 June to 25 June)	10	2.97	1.43, 5.47
Week 3 (26 June to 2 July)	7	2.08	0.84, 4.29
Week 4 (3 July to 9 July)	8	2.38	1.03, 4.69
Weeks 1–4 (12 June to 9 July)	39	11.60	8.25, 15.86
Weekly avg. (12 June to 9 July)	9.8	2.90	2.06, 3.96
Rimini (*n* = 2599)			
Week 1 (12 June to 18 June)	10	3.85	1.85, 7.08
Week 2 (19 June to 25 June)	12	4.62	2.39, 8.07
Week 3 (26 June to 2 July)	13	5.00	2.66, 8.55
Week 4 (3 July to 9 July)	7	2.69	1.08, 5.55
Weeks 1–4 (12 June to 9 July)	42	16.16	11.65, 21.84
Weekly avg. (12 June to 9 July)	10.5	4.04	2.91, 5.46

Notes: A total of six family doctors were included in the analysis (two in Ravenna, two in Forlì and two in Rimini). Abbreviations: CI, confidence interval.

**Table 3:** Symptoms observed among people aged >  14 years exposed to contaminated water during the 2023 Romagna floods from 16 May to 11 June vs. 12 June to 9 July, overall and by district of residence.

	16 May to 11 June 2023		12 June to 9 July 2023	
	Cases	Rate × 1000 Pop.	Cases	Rate × 1000 Pop.
		(95% CI)		(95% CI)
Skin infection				
All	17 *	0.80 (0.47, 1.28)	8	0.33 (0.14, 0.65)
Ravenna	7 *	1.10 (0.44, 2.26)	5	0.52 (0.17, 1.22)
Forlì	6	0.88 (0.32, 1.92)	1	0.15 (0.00, 0.82)
Cesena	4	0.96 (0.26, 2.46)	1	0.24 (0.01, 1.34)
Rimini	0	0.00 (0.00, 0.95)	1	0.26 (0.01, 1.44)
Diarrhea, vomiting or abdominal pain				
All	48 *	2.26 (1.67, 3.00)	26	1.06 (0.70, 1.56)
Ravenna	22 *	3.45 (2.16, 5.22)	19	1.98 (1.19, 3.10)
Forlì	13	1.91 (1.02, 3.26)	5	0.73 (0.24, 1.71)
Cesena	13	3.12 (1.66, 5.33)	0	0.00 (0.00, 0.88)
Rimini	0	0.00 (0.00, 0.95)	2	0.52 (0.06, 1.87)
Chest congestion, cough or suspected pneumonia				
All	26 *	1.23 (0.80, 1.79)	36	1.47 (1.03, 2.04)
Ravenna	11 *	1.73 (0.86, 3.09)	25	2.61 (1.69, 3.85)
Forlì	10	1.47 (0.70, 2.70)	5	0.73 (0.24, 1.71)
Cesena	5	1.20 (0.39, 2.80)	3	0.72 (0.15, 2.10)
Rimini	0	0.00 (0.00, 0.95)	3	0.78 (0.16, 2.27)
Conjunctivitis (Eye infection)				
All	18 *	0.85 (0.50, 1.34)	5	0.20 (0.07, 0.48)
Ravenna	10 *	1.57 (0.75, 2.88)	4	0.42 (0.11, 1.07)
Forlì	6	0.88 (0.32, 1.92)	1	0.15 (0.00, 0.82)
Cesena	2	0.48 (0.06, 1.73)	0	0.00 (0.00, 0.88)
Rimini	0	0.00 (0.00, 0.95)	0	0.00 (0.00, 0.95)
Otitis (Ear infection)				
All	13 *	0.61 (0.33, 1.05)	7	0.29 (0.12, 0.59)
Ravenna	6 *	0.94 (0.35, 2.05)	6	0.63 (0.23, 1.36)
Forlì	3	0.44 (0.09, 1.29)	0	0.00 (0.00, 0.54)
Cesena	4	0.96 (0.26, 2.46)	1	0.24 (0.01, 1.34)
Rimini	0	0.00 (0.00, 0.95)	0	0.00 (0.00, 0.95)
Fever (> 37.5 °C for ≥ 48 h)				
All	26 *	1.23 (0.80, 1.79)	12	0.49 (0.25, 0.86)
Ravenna	16 *	2.51 (1.43, 4.08)	7	0.73 (0.29, 1.50)
Forlì	9	1.32 (0.60, 2.51)	3	0.44 (0.09, 1.29)
Cesena	1	0.24 (0.01, 1.34)	0	0.00 (0.00, 0.88)
Rimini	0	0.00 (0.00, 0.95)	2	0.52 (0.06, 1.87)
Insomnia, agitation or anxiety				
All	36 *	1.70 (1.19, 2.35)	4	0.16 (0.04, 0.42)
Ravenna	20 *	3.14 (1.92, 4.84)	1	0.10 (0.00, 0.58)
Forlì	11	1.62 (0.81, 2.89)	3	0.44 (0.09, 1.29)
Cesena	5	1.20 (0.39, 2.80)	0	0.00 (0.00, 0.88)
Rimini	0	0.00 (0.00, 0.95)	0	0.00 (0.00, 0.95)

* Two family doctors from Ravenna with no available information were excluded (*n*  =  3209) Notes: A total of 16 family doctors were included in the analysis (six in Ravenna, four in Forlì, three in Cesena and three in Rimini). Abbreviations: CI, confidence interval.

**Table 4:** Symptoms observed among people aged > 14 years NOT exposed to contaminated water during the 2023 Romagna floods between 12 June and 9 July overall and by district of residence (12 June to 9 July 2023).

	Cases	Rate × 1000 Pop. (95% CI)
Skin infection		
All	13	1.42 (0.75, 2.42)
Ravenna	6	1.87 (0.69, 4.07)
Forlì	6	1.78 (0.65, 3.88)
Rimini	1	0.38 (0.01, 2.14)
Diarrhea, vomiting or abdominal pain		
All	22	2.40 (1.50, 3.63)
Ravenna	7	2.18 (0.88, 4.49)
Forlì	8	2.38 (1.03, 4.69)
Rimini	7	2.69 (1.08, 5.55)
Chest congestion, cough or suspected pneumonia		
All	52	5.67 (4.24, 7.44)
Ravenna	16	4.99 (2.85, 8.10)
Forlì	12	3.57 (1.84, 6.23)
Rimini	24	9.23 (5.92, 13.74)
Conjunctivitis (Eye infection)		
All	5	0.55 (0.18, 1.27)
Ravenna	2	0.62 (0.08, 2.25)
Forlì	1	0.30 (0.01, 1.66)
Rimini	2	0.77 (0.09, 2.78)
Otitis (Ear infection)		
All	9	0.98 (0.45, 1.86)
Ravenna	2	0.62 (0.08, 2.25)
Forlì	5	1.49 (0.48, 3.47)
Rimini	2	0.77 (0.09, 2.78)
Fever (> 37.5 °C for ≥ 48 h)		
All	35	3.82 (2.66, 5.31)
Ravenna	6	1.87 (0.69, 4.07)
Forlì	6	1.78 (0.65, 3.88)
Rimini	23	8.85 (5.61, 13.28)
Insomnia, agitation or anxiety		
All	1	0.11 (0.00, 0.61)
Ravenna	0	0.00 (0.00, 1.15)
Forlì	1	0.30 (0.01, 1.66)
Rimini	0	0.00 (0.00, 1.42)

Notes: A total of six family doctors were included in the analysis (two in Ravenna, two in Forlì and two in Rimini). Abbreviations: CI, confidence interval.

## Data Availability

All data are provided within the manuscript.

## Supplementary Materials

The following supporting information can be downloaded at: https://www.mdpi.com/article/10.3390/healthcare12171760/s1, Syndromic surveillance form.
