# Supplementary material for: Syndromic Surveillance in Public Health Emergencies: A Systematic Analysis of Cases Related to Exposure to 2023 Floodwaters in Romagna, Italy
Source: Healthcare (Basel). 2024 Sep 3;12(17):1760. doi: 10.3390/healthcare12171760 (PMC11395706; doi:10.3390/healthcare12171760)
Supplement: Supplementary file 1 [file healthcare-12-01760-s001.zip › healthcare-3180886-supplementary.pdf]

Sorveglianza della morbilità da calamità naturali
Per l'uso dei medici di assistenza primaria

Form v1

**Dr** _________________	*Malattia*	**Derm**	Infezioni cutanee primarie (es.erisipela,celluliti,inf.fungine) e infezioni secondarie di lesioni
**Città:**		**GI**	Diarrea / Vomito / Crampi addominali
**Settimana** dal______al______		**Resp**	Congestione / Tosse / Polmonite (anche sospetto)
		**Oft**	Congiuntivite
		**Oto**	Otite
**Totale** pazienti:		**Febbre**	Temperatura superiore a 37.5° per almeno 48 h
		**Neu**	insorgenza di insonnia, agitazione, ansia che ha richiesto uso di farmaci
Esposizione nei 14 giorni precedenti A= contatto con acque alluvionali<br>B= casa alluvionata<br>C= casa allagata per più giorni (>3)<br>D= coinvolgimento in attività di pulizia e ripristino<br>E= Nessuna esposizione	***PZ***		**NB** ogni riga corrisponde ad un paziente da indicare nella seconda colonna con le iniziali di nome e cognome (a.e. Mario Rossi: MR)

	PZ	Età (X)				Genere (X)		Esposizione (X)					*Malattia (X)*							
		<1	1-17	18-64	65+	M	F	A	B	C	D	E	Derm	GI	Resp	Oft	Oto	Febbre	Neu	Note
1																				
2																				
3																				
4																				
5																				
6																				
7																				
8																				
9																				
10																				
11																				
12																				
13																				
14																				
15																				
16																				
17																				
18																				
19																				
20																				
		TOTAL				TOTAL		TOTAL					TOTAL							

Inviare la scheda compilata: Ambito **Ravenna**: malattieinfettive.ra@auslromagna.it (tel.3343408988 08:30-13:30)/ | Ambito **Forlì**: profilassi.fo@auslromagna.it (tel. 3371000475 13.00-15.00) | Ambito **Cesena**: profilassi.ce@auslromagna.it tel 3371000475 (13.00-15.00) | Ambito **Rimini** : dipsan.rn@auslromagna.it e tel 0541707210-7211 08:30-13:30
